# Ethyl pyruvate reduces organic dust-induced airway inflammation by targeting HMGB1-RAGE signaling

**DOI:** 10.1186/s12931-019-0992-3

**Published:** 2019-02-06

**Authors:** Sanjana Mahadev Bhat, Nyzil Massey, Locke A. Karriker, Baljit Singh, Chandrashekhar Charavaryamath

**Affiliations:** 1 Department of Biomedical Sciences, 2008 Vet Med Building, Iowa State University, Ames, IA USA; 2 Department of Veterinary Diagnostic and Production Animal Medicine, 2203 Lloyd Veterinary Medical Center, Iowa State university, Ames, IA USA; 3 Faculty of Veterinary Medicine, 2500 University Dr. NW, University of Calgary, Calgary, T2N 1N4 Canada

**Keywords:** Organic dust, HMGB1, RAGE, Lung inflammation, Ethyl pyruvate

## Abstract

**Background:**

Animal production workers are persistently exposed to organic dust and can suffer from a variety of respiratory disease symptoms and annual decline in lung function. The role of high mobility group box-1 (HMGB1) in inflammatory airway diseases is emerging. Hence, we tested a hypothesis that organic dust exposure of airway epithelial cells induces nucleocytoplasmic translocation of HMGB1 and blocking this translocation dampens organic dust-induced lung inflammation.

**Methods:**

Rats were exposed to either ambient air or swine barn (8 h/day for either 1, 5, or 20 days) and lung tissues were processed for immunohistochemistry. Swine barn dust was collected and organic dust extract (ODE) was prepared and sterilized. Human airway epithelial cell line (BEAS-2B) was exposed to either media or organic dust extract followed by treatment with media or ethyl pyruvate (EP) or anti-HMGB1 antibody. Immunoblotting, ELISA and other assays were performed at 0 (control), 6, 24 and 48 h. Data (as mean ± SEM) was analyzed using one or two-way ANOVA followed by Bonferroni’s post hoc comparison test. A *p* value of less than 0.05 was considered significant.

**Results:**

Compared to controls, barn exposed rats showed an increase in the expression of HMGB1 in the lungs. Compared to controls, ODE exposed BEAS-2B cells showed nucleocytoplasmic translocation of HMGB1, co-localization of HMGB1 and RAGE, reactive species and pro-inflammatory cytokine production. EP treatment reduced the ODE induced nucleocytoplasmic translocation of HMGB1, HMGB1 expression in the cytoplasmic fraction, GM-CSF and IL-1β production and augmented the production of TGF-β1 and IL-10. Anti-HMGB1 treatment reduced ODE-induced NF-κB p65 expression, IL-6, ROS and RNS but augmented TGF-β1 and IL-10 levels.

**Conclusions:**

HMGB1-RAGE signaling is an attractive target to abrogate OD-induced lung inflammation.

## Background

Globally agriculture employs about 1.3 billion people [1] and in the US, about 1.8 million people work in the agriculture industry. However, agriculture is considered a dangerous profession due to significant number of deaths, injuries and morbidities (reviewed in [2]). People who work in agriculture and other related industries are persistently exposed to many contaminants and suffer from respiratory diseases and other conditions (reviewed in [2, 3]). Among the occupational contaminants, persistent exposure to organic dust (OD) is central to the negative health effects of work-related exposures. OD is a complex mixture of particulate matter of varying sizes, microbes and microbial products [4]. Workers from concentrated animal feeding operations (CAFOs) involved in swine, diary [5] and poultry production including duck hatcheries [6] (reviewed in [2]) as well as other industries (sewage handlers, waste handlers and bakery workers) are persistently exposed to the OD.

In North America, more than one million men, women and children suffer from exposure to OD [7]. There is a strong link between exposure to OD and development of inflammatory airway diseases and annual decline in lung function (reviewed in [8, 9]). Individuals exposed to OD report a range of respiratory and other symptoms including bronchitis, chest tightness, nasal congestion, organic dust toxic syndrome, occupational asthma, mucus membrane irritation, nausea, headache, mood changes, altered immunity [10–12], and increased risks of lung cancer [13]. Presence of multiple microbial and non-microbial factors in OD and broad-range of health effects upon exposure are a significant public health concern. Currently, there are limited therapeutic options to treat OD-induced inflammatory airway diseases.

OD exposure induced respiratory symptoms and long-term changes in lung function are a major occupational health issue in swine production workers (reviewed in [2, 4]). Previously, experimental exposure of human volunteers to swine barn environment indicated an underlying inflammatory process [14]. We employed a unique rat model mimicking human occupational exposure to swine barn environment and showed that single 8 h exposure induces airway inflammation and reactivity. In the same model, multiple (5 and 20-day) exposures resulted in dampened airway inflammation and reactivity. Surprisingly, there was an increase in the number of mucus producing goblet cells in the airways and activation of bronchus-associated lymphoid tissue (BALT) following 20-day exposure [15]. Mechanisms leading to the development of these airway remodeling features despite dampened inflammation and airway reactivity with 20-day exposure remain elusive.

Next, using wild type or *tlr4* mutant mouse, we demonstrated that barn exposure-induced lung inflammation, but not airway reactivity, is dependent on TLR4. In the same model, we documented airway epithelial damage in a TLR4-independent manner [16]. Subsequently, the roles of TLR9 [17], TLR2 [18], NOD2 [19], MyD88 [20], and protein kinase C epsilon (PKC ε) in organic dust-induced airway inflammation have been demonstrated. OD exposure has also been linked to bone loss indicating the systemic effects of exposure [21] (reviewed in [22]). These studies and our previous work (reviewed in [2]) demonstrate that OD is complex in composition and inhaled OD elicits host response through multiple signaling pathways. Despite increased understanding of mechanisms of OD-induced lung inflammation, therapeutic options to treat OD-induced lung diseases are limited.

Damage associated molecular patterns (DAMPs) are endogenous molecules that are released upon tissue damage [23]. DAMPs are increasingly becoming important in chronic airway diseases [24]. High-mobility group box 1 (HMGB1) is a prototype DAMP present in almost all nucleated cells. HMGB1 is a normal nuclear protein that upon translocation to cytoplasm and secretion into extracellular milieu behaves as a DAMP with inflammatory cytokine-like properties (reviewed in [25, 26]). Immune activation or necrosis is known to cause nucleocytoplasmic translocation and release of HMGB1 into extra-cellular space in many inflammatory airway diseases [24, 26]. HMGB1 is known to play a pathogenic role in asthma with contributions to airway smooth muscle (ASM) dysfunction and airway reactivity [27]. Blocking HMGB1 has been beneficial in a mouse model of allergic airway disease and sepsis [28, 29].

Post-translational modifications such as phosphorylation and acetylation determine the nucleocytoplasmic translocation, secretion and pathogenic role of secreted HMGB1 [30, 31]. Nucleocytoplasmic translocation of HMGB1 involves JAK-STAT1 mediated acetylation of lysine residues on nuclear localization sites (NLS) whereas pyroptosis or exocytosis of secretory lysosomes leads to secretion of HMGB1 into extracellular milieu (reviewed in [32]). Several tools such as JAK/STAT1 inhibitor [33], sirtuin 1 [34], anti-HMGB1 antibodies [35] and ethyl pyruvate [36] have been used to abrogate the pathological effects of HMGB1.

We tested a hypothesis that OD exposure of airway epithelial cells induces translocation of HMGB1 and blocking HMGB1 translocation dampens OD-induced lung inflammation. In the current study, using a human airway epithelial cell line (BEAS-2B) model, we demonstrate that OD-exposure induces nucleocytoplasmic translocation of HMGB1 and inflammation. Further, we show that EP or anti-HMGB1 treatment reduces OD-induced airway inflammation via blocking HMGB1 translocation and signaling through secreted HMGB1 respectively.

## Methods

### Rats and organic dust exposure

Rat model of organic dust exposure has previously been described [15]. Rat exposure to the swine barn environment (organic dust exposure) was conducted with approved protocols from University of Saskatchewan Campus Committee on Animal Care. All the animal experiments were performed as per the Canadian Council on Animal Care Guidelines. Six-week-old, male, Sprague-Dawley rats (*n* = 5/group, Charles River Laboratories) were exposed to either ambient air (control) or one, five or 20-days to swine barn environment (8 h/day). At the end of the exposure period, rats were euthanized, lung tissues were collected and processed for immunohistochemistry [15]. The paraformaldehyde-fixed, paraffin-embedded tissues from these rats were used in the current study.

### Immunohistochemical analysis

Immunohistochemistry on five-micron thick tissue sections (*n* = 5 rats/group) was performed using anti-HMGB1 (1:1000, Abcam) and HRP (1:1000, ant-rabbit IgG; Abcam) and counterstained with methyl green (Vector Laboratories, Inc., Burlingame, CA). An investigator blinded to the treatment groups semi-quantified the cell specific expression of HMGB1 in bronchioles, endothelium of blood vessels, alveolar septa, ASM and bronchus associated lymphoid tissue (BALT) using predetermined scoring criteria (outlined in Table 1). Scored regions were photographed (Nikon Eclipse TE2000-U; Spot Advance imaging software, Michigan, USA).

**Table 1:** Semi-quantitative evaluation of HMGB1 expression and criteria for sassigning scores

Score	Criteria
0	No expression
1	Minimal
2	Mild
3	Moderate
4	Intense

### Organic dust extract preparation

Settled dust samples from typical swine housing facilities (representing organic dust) were collected into zip lock bags and transported on ice and stored at − 80 °C until processed. A sterile organic dust extract was prepared as per a published protocol [37]. Dust samples were weighed and for every gram of dust, 10 mL of Hanks’ balanced salt solution without calcium (Gibco) was added, stirred and allowed to stand at room temperature for an hour. The mixture was centrifuged (1365 x g, 4 °C) for 20 min, supernatant recovered, and pellet was discarded. Supernatant was centrifuged again with same conditions, pellet discarded and recovered supernatant was filtered using 0.22 μm filter and stored at − 80 °C until used. The filter sterilized organic dust extract (ODE) samples were considered 100% and diluted to 1–5% (*v*/v) before use in experiments.

### Endotoxin estimation

The levels of endotoxin in the ODE samples was quantified using the Pyrochrome® chromogenic endotoxin assay kit (Associates of Cape Cod, Inc., East Falmouth, MA). The ODE samples were diluted in a ratio of 1:10 in endotoxin free water. The samples along with reconstituted pyrochrome lysate, were added to a 96-well plate in a sample to lysate ratio of 1:4. The standard was reconstituted as per manufacturer’s recommendation and added to the plate in a sample to lysate ratio of 1:4. The microplate was incubated at 37 °C with shaking and the absorbance was read at 405 nm (every 10 min, three readings over a total of 30 min) using the Gen 5™ software in BioTek® ELx808™ spectrophotometer.

### Cell culture and treatments

Immortalized human bronchial epithelial cells (BEAS-2B, ATCC CRL-9609) have previously been used to study innate inflammatory responses to ODE [38, 39]. BEAS-2B cells were seeded onto type I bovine collagen (StemCell Technologies, Vancouver, BC, Canada) coated T-75 flasks. Cells were grown submerged in serum free LHC-9 medium (Gibco) containing 100 U/mL of Penicillin/Streptomycin (Gibco) and 2 μg/mL of Amphotericin B (Sigma) in a humidified chamber with 5% CO_2_ at 37 °C until approximately 80% confluence was achieved.

**Fig. 1:**
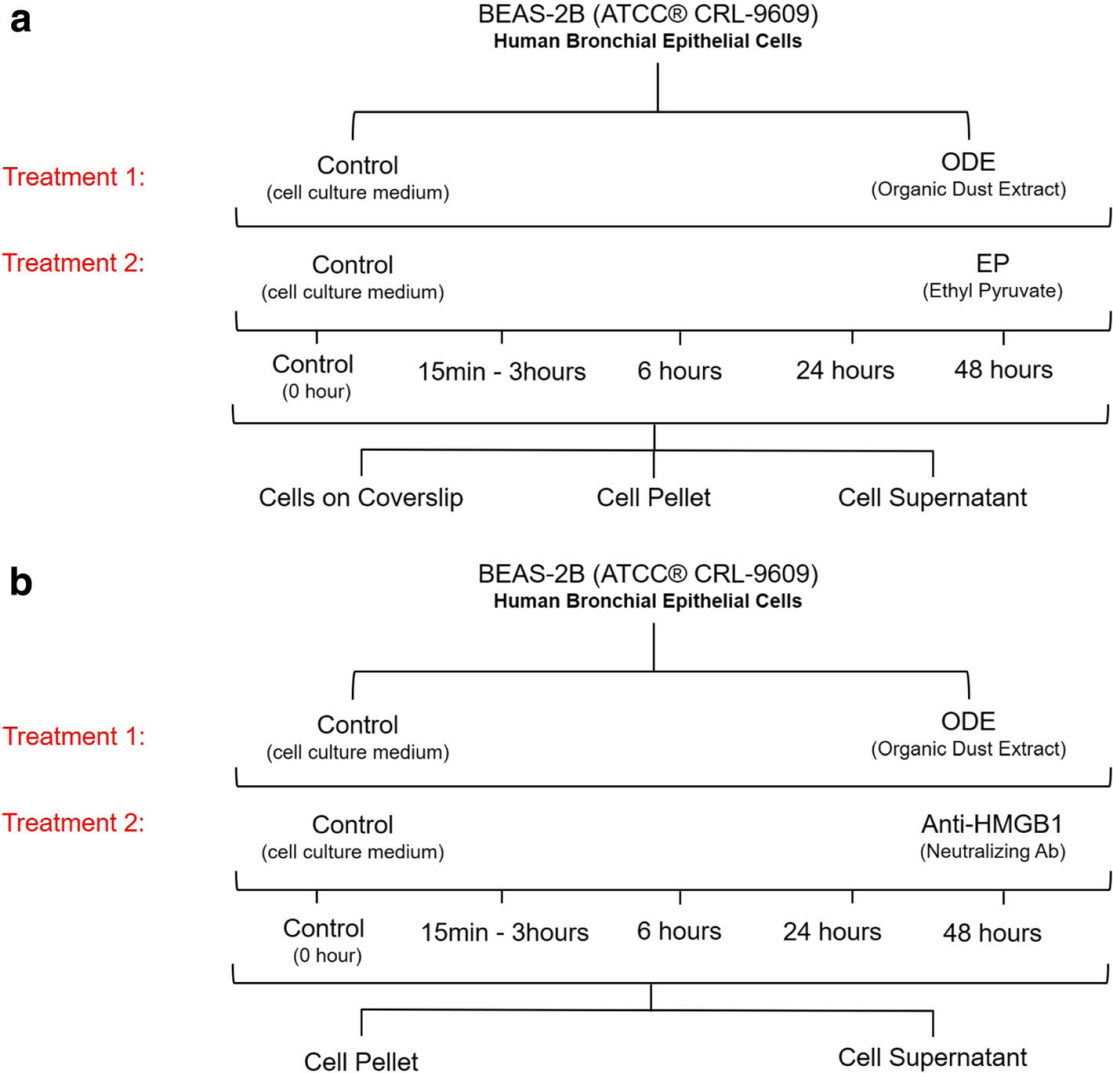
ODE exposure of BEAS-2B cells and EP or anti-HMGB1 neutralizing antibody treatment. BEAS-2B cells were treated with either media (control) or ODE (treatment 1) followed by either media, EP (**a**, treatment-2) or neutralizing HMGB1 antibody (**b**, treatment 2). Cells were processed for various assays at 0 (control), 6, 24 and 48 h by collecting cells on coverslips or cell pellet or cell supernatant

Ethyl pyruvate (EP, Santa Cruz Biotechnology, CA) was reconstituted in Ringer’s solution (Sigma-Aldrich, St. Louis, MO, USA) and used at a final concentration of 2.5 μM in the cell culture medium (Fig. 1). Neutralization of secreted HMGB1 was carried out using anti-HMGB1 antibody (BioLegend, CA) at a concentration of 10 μg/mL [40].

Our in vitro model of ODE exposure and treatments are represented (Fig. 1 a and b). Cells were treated with either medium (control) or lipopolysaccharide (LPS, *Escherichia coli* O127:B8; Sigma) or peptidoglycan (PGN, *Staphylococcus aureus*; Sigma), or ODE (1–5% *v*/v) (treatment 1, Fig. 1) followed by a co-treatment with either medium or EP (treatment 2, Fig. 1a) or anti-HMGB1 neutralizing antibody (treatment 2, Fig. 1b). Following treatment, 1 and 2, samples were processed at 0, 6, 24 and 48 h for various assays. For NF-κB p65 assays, samples were collected at 15 and 30 min as well as 1, 1.5, 2 and 3 h. Control samples did not differ at various time points (6, 24 and 48 h) and hence controls samples at 0 h were included for all data analysis.

LPS and PGN were used as standard pathogen associated molecular patterns (PAMPs) (data not shown). Table 2 summarizes the stock and working concentrations of various PAMPs used in treatment 1 (Fig. 1) prepared by dissolving in LHC-9 medium from stock concentrations.

**Table 2:** Stock and working concentrations of cell treatments

Treatments	Stock concentration (in cell culture grade water)	Working concentration (in LHC-9)
LPS	5 mg/mL	10 μg/mL
PGN	1 mg/mL	10 μg/mL
ODE	100%	1–5%

### Cell viability assay

Prior to conducting experiments, cell viability was assessed. Live/dead cell count was determined by 4% trypan blue dye (EMD Millipore, Burlington, MA) exclusion and percentage viability was calculated. Population of cells with more than 95% viability were used for the experiments.

### Immunofluorescence microscopy

Cells were seeded (1 × 10^6^ cells/well) in 12-well plates on Poly D-lysine hydro bromide (Sigma-Aldrich, St. Louis, MO) coated cover slips and exposed to the treatments as outlined in Fig. 1. Cells were fixed with 4% paraformaldehyde in PBS for 20 min at room temperature and washed. Cells were blocked for an hour using a blocking buffer containing 10% normal donkey serum (EMD Millipore, Burlington, MA), 0.2% triton X 100 and PBS. Cover slips with cells were incubated with anti-HMGB1 (1:1000 dilution, rabbit polyclonal) and anti-RAGE (1:200 dilution, rabbit polyclonal) antibodies in antibody diluent solution (2.5% normal donkey serum, 0.25% sodium azide, 0.2% triton X 100, PBS) (AbCam, Cambridge, MA) with overnight at 4 °C. Next, coverslips were incubated with donkey anti-rabbit biotin conjugated secondary antibody (1:400, diluted in antibody diluent, HMGB1) and 1:500 dilutions of FITC (RAGE) (Jackson Immunoresearch, West Grove, PA) for an hour at room temperature, followed by streptavidin-Cy3 (1:300 in PBS, HMGB1). Coverslips were mounted onto slides using VECTASHIELD antifade mounting medium with 4′,6-Diamidino-2-Phenylindole, Dihydrochloride (DAPI, Vector Labs, Burlingame, CA) and imaged using Axiovert 200 M Zeiss inverted fluorescence microscope (Zeiss, Deutschland, Germany) equipped with Hamamatsu camera. Images were processed using HCImage live 4 software (Hamamatsu Corporation, Sewickley, PA).

### Western blot analysis

Cells were harvested followed by separation of cytoplasmic and nuclear fractions using NE-PER nuclear and cytoplasmic extraction kit supplemented with a mixture of protease and phosphatase inhibitors (Thermo Scientific, USA). For NF-κB p65 detection, whole cell lysates were used as well. Total protein levels were estimated by Bradford assay and equal amounts of protein (20 μg/sample), along with a molecular weight marker (Bio-Rad, Hercules, CA), were loaded on to 12% Tris-glycine gels (Bio-Rad, Hercules, CA). The gels were subjected to 100 V for 1–2 h at 4 °C. Next, proteins were transferred on to a nitrocellulose membrane at 23 V at 4 °C for 16 h. Membranes were washed once with distilled water and non-specific binding was blocked with fluorescent western blot blocking buffer (Rockland Immunochemicals, PA, USA) in PBS at room temperature for an hour. Membranes were washed twice with 1X PBS with 0.05% tween 20 (PBST) and incubated with primary rabbit monoclonal anti-HMGB1 antibody (1:1000 dilution) or rabbit polyclonal anti-NF-κB p65 antibody (1:1000 dilution), mouse polyclonal anti-β-Actin (1:6000) and rabbit polyclonal anti-Lamin B1 (1:1000) (AbCam, Cambridge, MA) antibodies overnight at 4 °C. β-actin (whole cell lysate and cytoplasmic fractions) and Lamin-B1 (nuclear fraction) were used as loading controls. Membranes were then washed and incubated with goat anti-mouse and donkey anti-rabbit IgG P680 (1:10,000 dilution) secondary antibody (Thermo-Scientific, USA). Membranes were scanned using the Odyssey® CLx IR imaging system (LI-COR Biotechnology, Lincoln, NE) and analysis was performed using ImageJ program (National Institute of Health).

### Measurement of reactive oxygen species

Intracellular reactive oxygen species (ROS) production was measured using chloromethyl derivative of dichlorodihydrofluorescein diacetate (CM-H_2_DCFDA) (ThermoFisher Scientific, USA). A working solution of 10 μM of DCFDA in PBS was used. BEAS-2B cells (5 × 10^4^/well) were seeded in a 96 well cell culture plate and incubated in a 5% CO_2_ incubator to reach confluence. The cells were incubated with H_2_DCFDA working solution at 37 °C for 30 min, followed by treatments as outlined in Fig. 1. The fluorescence intensity of the oxidized form of H_2_DCFDA was measured at excitation/emission wavelengths of 488/535 nm (SpectraMax M2 Gemini Molecular Device Microplate Reader). The results were expressed as percentage fluorescence relative to control.

### Griess assay

Griess assay was performed as described [41]. Briefly, nitric oxide secretion was measured (representing reactive nitrogen species (RNS)) as nitrite levels in cell culture media using Griess reagent (Sigma) and sodium nitrite standard curve, prepared using a stock solution of 200 μM. The assay was performed in a 96 well-plate and absorbance was measured at 550 nm (SpectraMax M2 Gemini Molecular Device Microplate Reader). The results were expressed as μM concentration of nitrite secreted.

### Cytokine analysis

GM-CSF, IL-1β, IL-8, IL-6, IL-10 and TGF-β1 levels in BEAS-2B cell culture supernatant were measured using ELISA kits (ThermoFisher Scientific, USA) in accordance with the manufacturer’s recommendations.

#### qRT-PCR

RNA was isolated using TRIzol extraction methods [42] and RNA concentration was measured using NanoDrop spectrophotometer. Two micrograms of RNA was used to synthesize cDNA using the Superscript III first strand synthesis kit (ThermoFisher Scientific, USA) following the manufacturer’s protocol. For qPCR reactions, 5 μL of SYBR Green Mastermix (ThermoFisher Scientific, USA), 1 μL of primers, 1–2 μL of water and 1–2 μL of cDNA was used. The primers for genes of interest (Table 3) were synthesized at Iowa State University’s DNA Facility. The housekeeping gene 18 S rRNA (ThermoFisher Scientific, USA) was used in all qPCR reactions. No-template controls and dissociation curves were run for all reactions to exclude cross-contamination. The qRT-PCR reactions were run in a Bio-Rad CFX Connect™ detection system and the data was analyzed using 2^-ΔΔCT^ method [43].

**Table 3:** Primer sequences used for qRT-PCR

Gene Symbol	Primer Sequence (5′ **→** 3′)	
*nfκbp65*	Forward	CCAGACCAACAACAACCCCT
	Reverse	TCACTCGGCAGATCTTGAGC
*nfκbp50*	Forward	GCAGCACTACTTCTTGACCACC
	Reverse	TCTGCTCCTGAGCATTGACGTC
*nfκbp52*	Forward	GGCAGACCAGTGTCATTGAGCA
	Reverse	CAGCAGAAAGCTCACCACACTC
*relB*	Forward	TGTGGTGAGGATCTGCTTCCAG
	Reverse	TCGGCAAATCCGCAGCTCTGAT
*crel*	Forward	AGTTGCGGAGACCTTCTGACCA
	Reverse	CGTGATCCTGGCACAGTTTCTG
*tlr2*	Forward	CTTCACTCAGGAGCAGCAAGCA
	Reverse	ACACCAGTGCTGTCCTGTGACA
*tlr4*	Forward	CCCTGAGGCATTTAGGCAGCTA
	Reverse	AGGTAGAGAGGTGGCTTAGGCT

#### Statistical analysis

Data were expressed as mean ± SEM and analyzed by one-way or two-way ANOVA followed by Bonferroni’s post hoc comparison tests (GraphPad Prism 7.0, La Jolla, CA, USA). A *p*-value of < 0.05 was considered statistically significant.

## Results

### Barn exposure and HMGB1 expression in the lungs

**Fig. 2:**
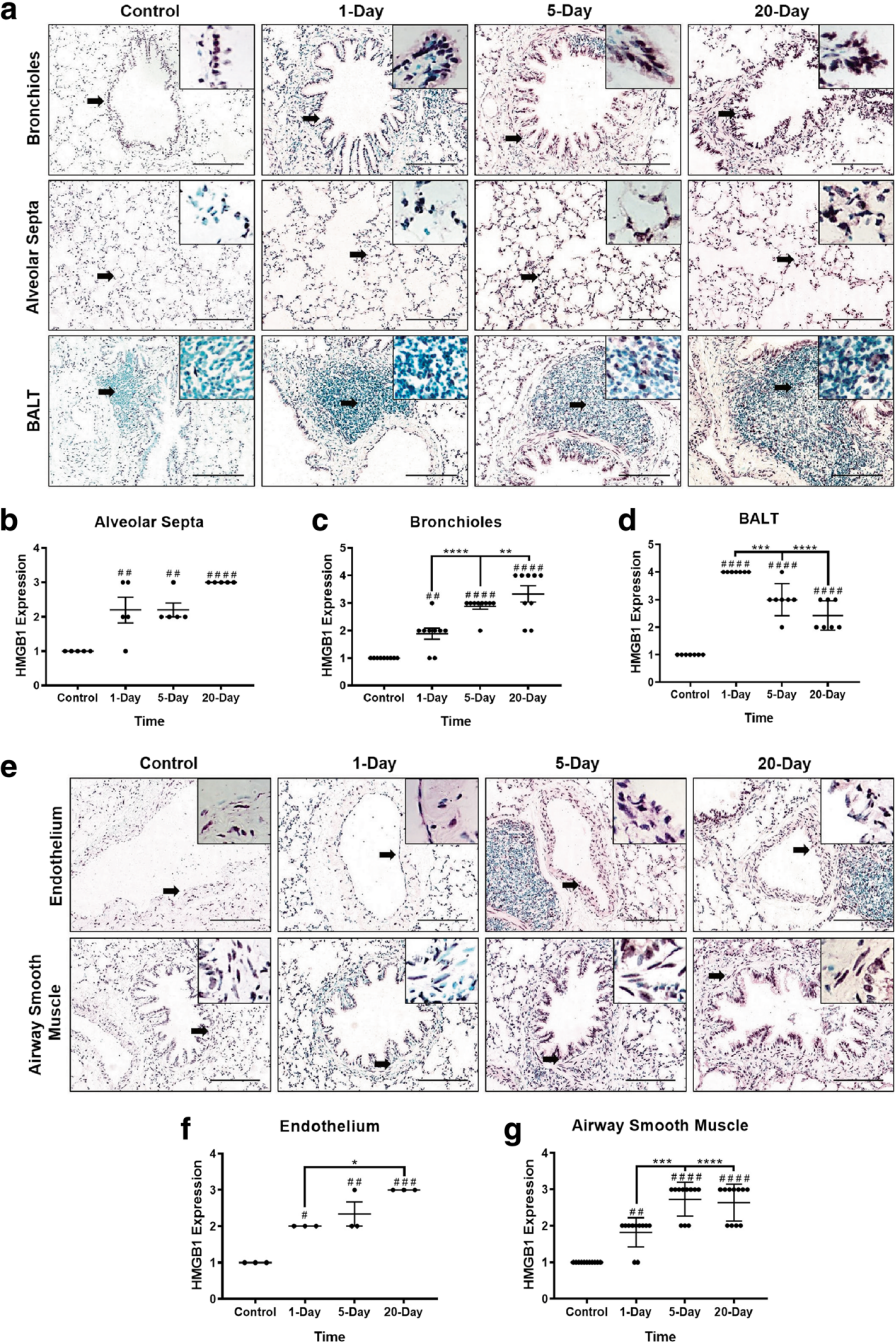
OD exposure of rats in the swine barn work environment and HMGB1 expression. Immunohistochemical staining for HMGB1 expression was performed on rat lung tissues. Compared to controls, one, five and 20-day barn (organic dust) exposure of rats induced an increase in the expression of HMGB1 in the bronchioles, alveolar septa and BALT (**a**) and endothelium of the blood vessels and ASM (arrows and inset, bar = 100 μm, **e**), respectively. One-way ANOVA performed on immunohistochemical scores for HMGB1 expression in bronchioles (9 fields/animal), alveolar septa (5 fields/animal) and BALT (7 fields/animal) (**b**-**d**) and endothelium of blood vessels (3 fields/animal) and ASM (11 fields/animal) is presented (**f** and **g**). *p < 0.05, **p < 0.005, ***p < 0.0005, ****p < 0.0001

Using immunohistochemistry, we delineated cell specific semi-quantitative expression of HMGB1 in control and barn exposed rat lungs. The overall staining was assigned scores in a blinded manner by using a predetermined criterion (Table 1). Compared to controls, barn-exposed rat lungs showed significantly higher expression of HMGB1 in bronchiolar epithelium, alveolar septa, BALT, endothelium of blood vessels and ASM (Fig. 2, a-g). In the bronchiolar epithelium, HMGB1 expression was at the tip of the airway epithelium. In the alveolar septa, type 2 alveolar epithelial cells showed predominant staining. Type 1 alveolar epithelial cells and alveolar macrophages were stained as well. Since these rat lungs had previously been lavaged, alveolar macrophages were not in abundance.

### Endotoxin content of OD samples

Endotoxin units (EU/mL) measured in OD samples are presented (Table 4).

**Table 4:** Endotoxin assay to measure LPS content in the ODE samples

Sample No.	LPS (EU/mL)
1	1.140 ± 0.001
2	0.990 ± 0.0005
3	1.337 ± 0.0006
4	1.433 ± 0.02
5^a^	1.417 ± 0.002
6^b^	0.8067 ± 0.0008
7^c^	1.263 ± 0.0008

^a^Pooled sample from samples 1 and 2

^b^Pooled sample from samples 3 and 4

^c^Pooled sample from samples 1, 2, 3 and 4

### EP treatment reduces ODE induced cytoplasmic expression of HMGB1

**Fig. 3:**
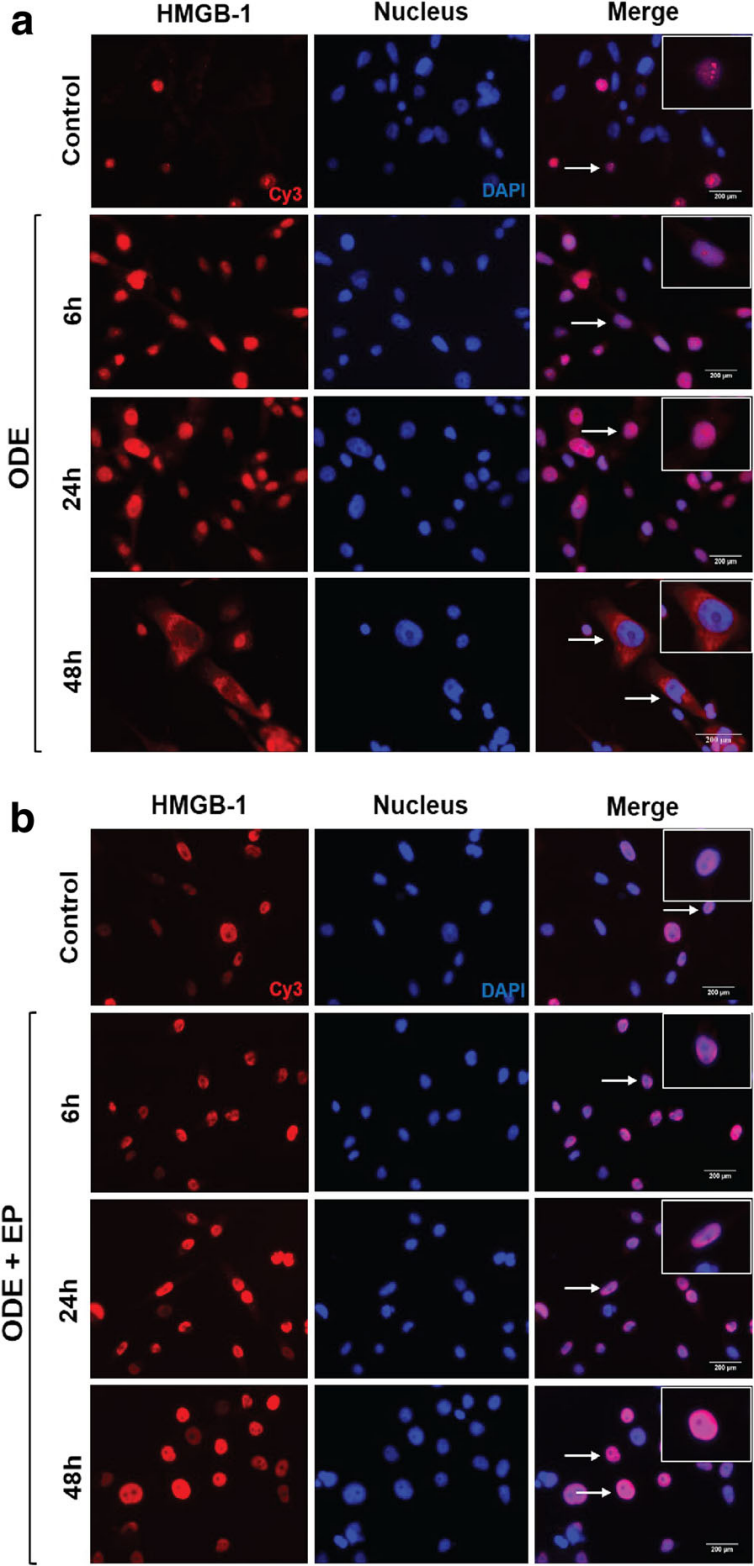
EP reduces ODE-exposure induced nucleocytoplasmic translocation of HMGB1. Medium (control, 0 h) or ODE (6, 24 and 48 h post) treated cells were stained with polyclonal anti-HMGB1 antibody and DAPI stain delineated the nuclei. Compared to controls, ODE treated cells showed nucleocytoplasmic translocation of HMGB1 (arrows and inset, bar = 200 μm, **a**). Compared to vehicle (medium), co-treatment with EP (2.5 μM) showed a marked decrease in ODE-induced nucleocytoplasmic translocation of HMGB-1 (arrows and inset, micrometer = 200 μm, **b**)

We used immunocytochemistry to detect expression of HMGB1. Using DAPI-stained nuclei as a reference nucleocytoplasmic translocation of HMGB1 was identified. Compared to the medium-treated controls, ODE-treated cells (48 h) showed increased expression of HMGB1 in the cytoplasm (arrow in merged image, Fig. 3 a). Compared to vehicle (medium), co-treatment with EP abrogated the ODE-induced increased expression of HMGB1 in the cytoplasm (arrow in merged image, Fig. 3 b).

### EP treatment reduces ODE induced HMGB1 expression in the cytoplasmic fraction

**Fig. 4:**
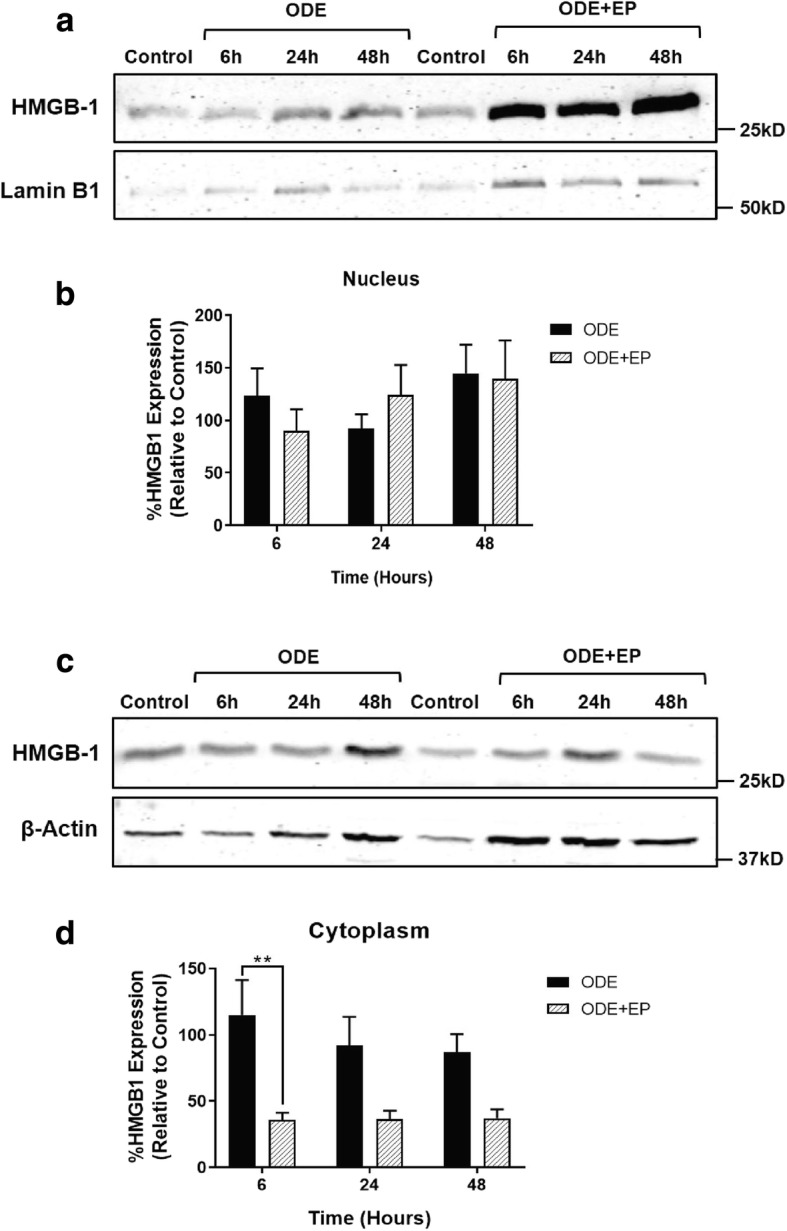
EP reduces ODE-exposure induced nucleocytoplasmic translocation of HMGB1. Medium (control, 0 h) or ODE (6, 24 and 48 h post) treated cells were processed for separation of nuclear and cytoplasmic fractions and western blotting to detect HMGB1 protein. Compared to controls, ODE treated cells showed a temporal increase in HMGB1 expression (25 kD) in the (**a**) nuclear fraction at 6 and 48 h. Compared to vehicle (Ringer’s solution), co-treatment with EP (2.5 μM) resulted in significantly decreased levels of HMGB1 in the cytoplasm at 6 h post-treatment indicating reduction in ODE-induced nucleocytoplasmic translocation of HMGB1 (**d**). HMGB1 (25kD) bands were normalized over either Lamin B1 (50kD, cytoplasmic fraction, a) or β-actin (37kD, nuclear fraction, **b**) and percentage intensity (*n* = 5/group) of treatment groups relative to control were analyzed using two-way ANOVA (**c** and **d**). **p < 0.01 (* indicates difference within the OD/barn exposure groups)

We performed western blotting to quantify the expression of HMGB1 in nuclear and cytoplasmic fractions of ODE-treated cells. Compared to controls, ODE exposure increased the HMGB1 protein levels in nuclear (6 and 48 h) and cytoplasmic (6 h) fractions (Fig. 4, a-d). Compared to the vehicle (medium), co-treatment with EP significantly reduced the ODE-induced increase in HMGB1 protein levels in the cytoplasmic fractions at 6 h (Fig. 4, b and d).

### ODE exposure results in HMGB1 and RAGE co-localization

**Fig. 5:**
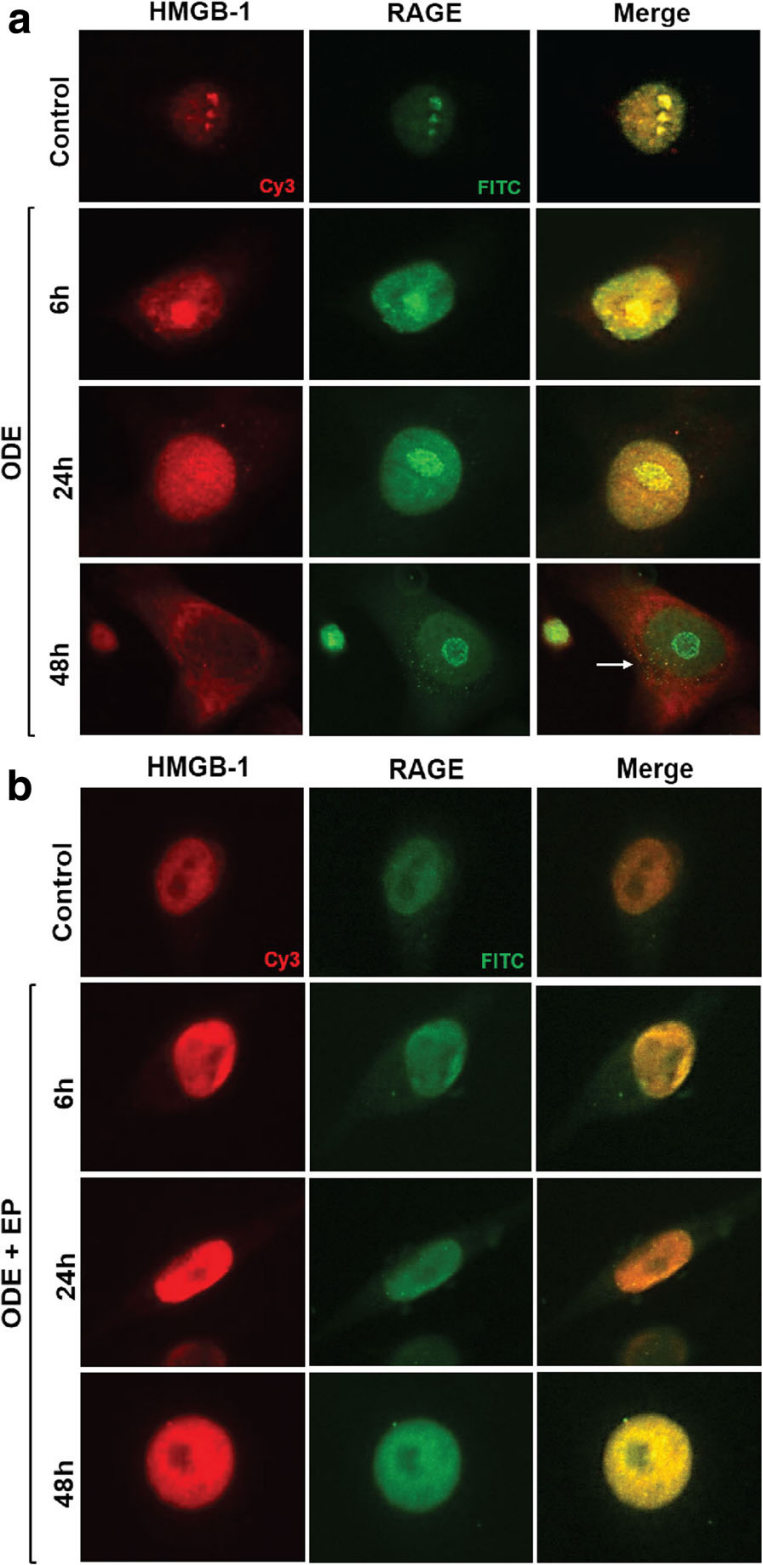
EP reduces ODE-exposure induced augmentation of RAGE expression and HMGB1-RAGE co-localization in the cytoplasm. Medium (control, 0 h) or ODE treated (6, 24 and 48 h post) treated cells were stained with polyclonal anti-HMGB1 or anti-RAGE antibodies. Compared to controls, ODE treated cells showed increased expression and nucleocytoplasmic translocation of HMGB1 (arrowhead, 48 h, **a**), increased expression of RAGE (48 h, **a**) and co-localization of HMGB1 and RAGE (white arrows, 48 h, **a**). Compared to vehicle (Ringer’s solution), co-treatment with EP (2.5 μM) resulted in a marked decrease in ODE-induced nucleocytoplasmic translocation of HMGB-1 and co-localization of HMGB1 and expression of RAGE (arrows and inset, bar = 200 μm, **b**)

We performed immunocytochemistry for evaluating the expression of HMGB1 and RAGE. Following cell treatments, anti-HMGB1 and anti-RAGE stained (Cy3 and FITC, respectively) images were merged. Compared to controls (medium treated, 0 h), ODE-treatment induced an increase in the expression and co-localization of HMGB1 and RAGE in the cytoplasm (arrow, Fig. 5a, 48 h); both changes were inhibited by co-treatment with EP. Following EP treatment, HMGB1 remained arrested in the normal nuclear location (Fig. 5b, 48 h).

### ODE induced ROS and RNS production

**Fig. 6:**
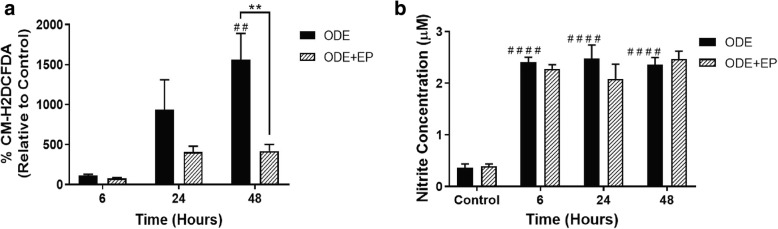
ODE exposure induces ROS and nitrite (secreted RNS) production and EP treatment reduces ODE-induced ROS production. Media alone (control, 0 h) or ODE (6, 24, and 48 h) treated cells were subjected to CM-H2DCFDA and Griess’ assay to quantify intracellular ROS production (**a**) and secreted nitrite concentration (**b**) respectively (*n* = 6). ODE exposure of cells resulted in significant increase in intracellular ROS and nitrite secretion (secreted RNS) into the media as early as 6 h post-treatment. Compared to vehicle treatment, cells co-treated with EP (2.5 μM) showed a significant reduction in ODE-induced ROS production at 48 h. Data analyzed with two-way ANOVA is represented (**a** and **b**). ## or **p < 0.01 and **** or #### *p* < 0.0001. # indicates significantly different from control whereas * indicates significant difference within the OD/barn exposure groups

We quantified ROS and RNS levels in control and ODE treated cells to understand the effect of exposure to ODE in the presence or absence of HMGB1 secretion (EP treatment). Compared to the controls (medium, 0 h), ODE-treated cells produced significantly higher amounts of ROS and RNS at 6, 24, and 48 h (Fig. 6, a and b). Compared to vehicle (medium), co-treatment with EP significantly reduced ODE-induced ROS (Fig. 6a) but not the RNS production (Fig. 6b).

### ODE induced pro-inflammatory cytokines production

**Fig. 7:**
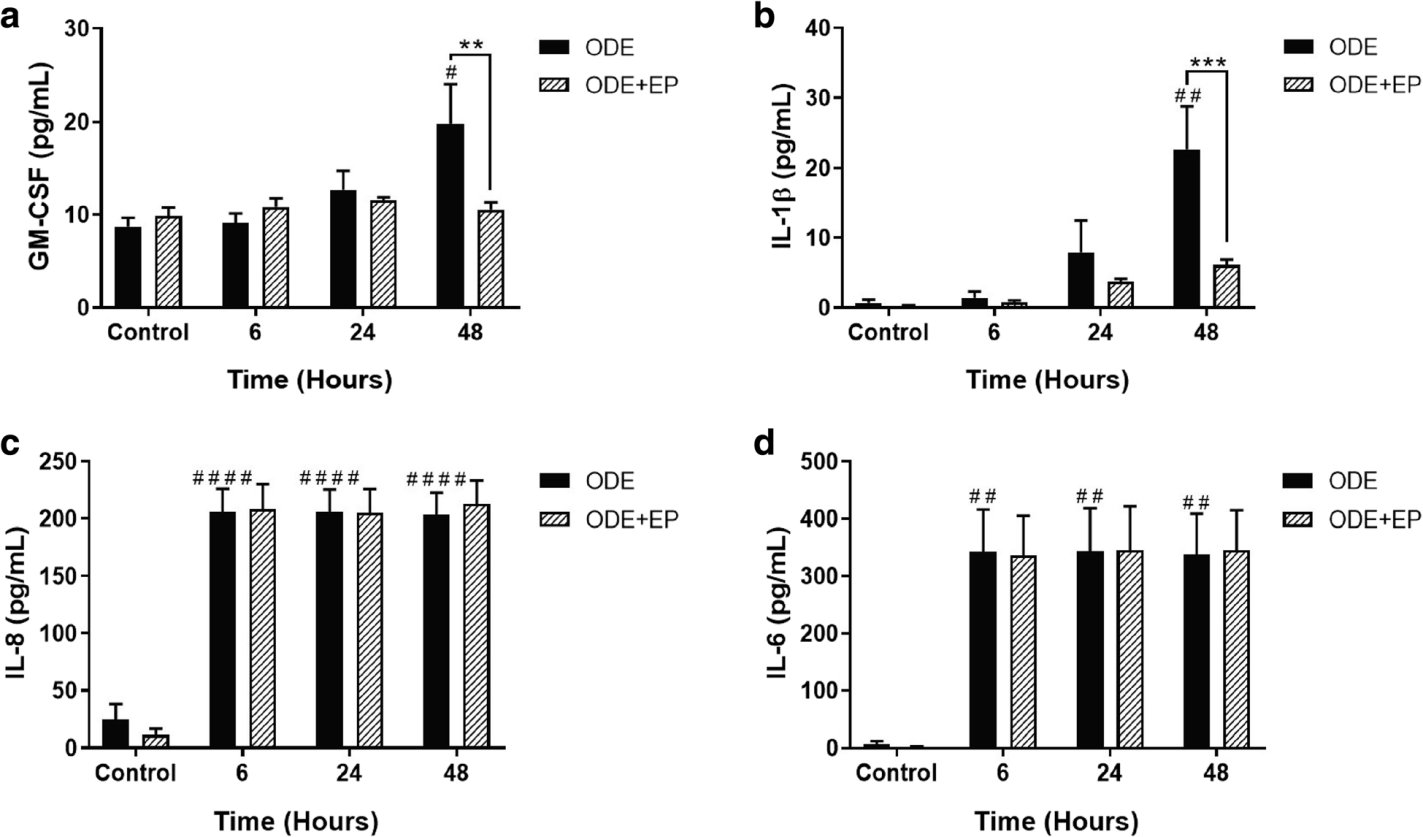
EP treatment reduces ODE exposure induced secretion of GM-CSF and IL-1β but not IL-8 and IL-6 levels. Compared to medium (controls, 0 h), ODE treated BEAS-2B cells secreted increased levels of GM-CSF, IL-1β, IL-8 and IL-6 (**a**-**d**) respectively. Compared to vehicle (Ringer’s solution), co-treatment with EP (2.5 μM) significantly reduced ODE-induced GM-CSF and IL-1β levels (**a** and **b**). Data (*n* = 6) analyzed using two-way ANOVA is represented. * or # *p* < 0.05, ** or ## *p* < 0.01, *** or ### *p* < 0.001, **** or #### *p* < 0.0001. # indicates different from control whereas * indicates difference within the OD/barn exposure groups

We quantified various cytokines in treated cell-supernatants to understand the effect of ODE in the presence or absence of HMGB1 secretion (EP treatment). Compared to controls (medium, 0 h), ODE-treated cells produced significantly higher amounts of GM-CSF, IL-1β, IL-8 and IL-6 (*p* < 0.05, Fig. 7, a-d). Compared to vehicle (medium), co-treatment with EP significantly reduced ODE-induced increase in GM-CSF and IL-1β (Fig. 7, a and b respectively) but not IL-8 and IL-6 levels.

### EP-treatment increases ODE-induced production of TGF-β1 and IL-10

**Fig. 8:**
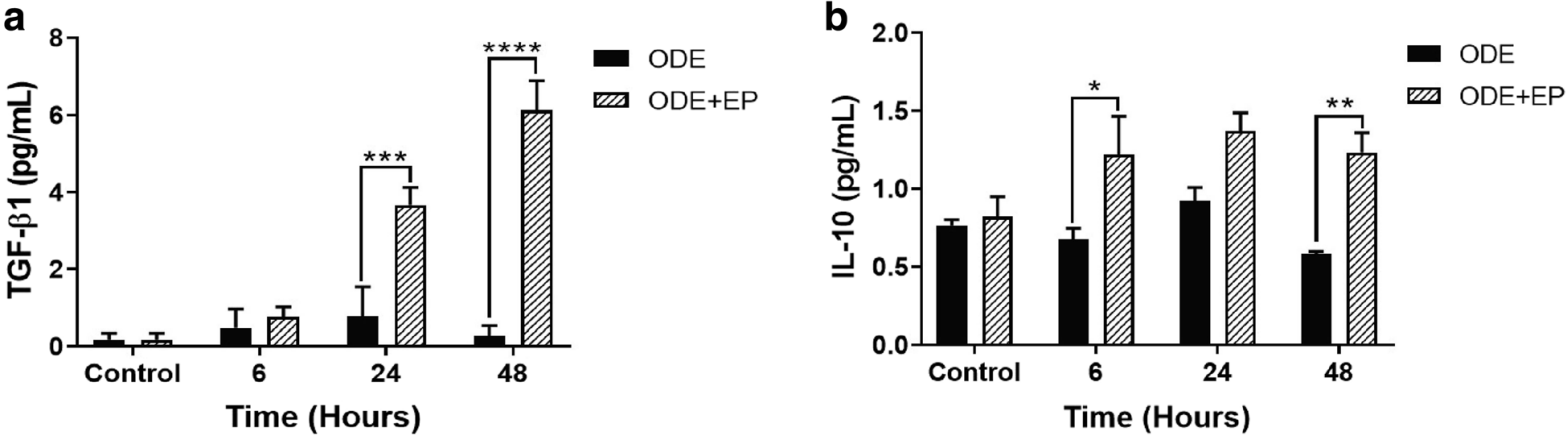
EP-treatment augments ODE-induced production of TGF-β1 and IL-10 levels in BEAS-2B cells. Compared to medium (control, 0 h), co-treatment of ODE exposed BEAS-2B cells with EP (2.5 μM) significantly increased the production of TGF-β1 (24 and 48 h) and IL-10 (6, 24 and 48 h).d Data (*n* = 6) analyzed with two-way ANOVA is represented. * or #*p* < 0.05, ** or ##*p* < 0.01, *** or ### *p* < 0.001, **** or #### *p* < 0.0001. # indicates different from control whereas * indicates difference within the OD/barn exposure groups

Compared to controls (medium, 0 h), ODE-treated cells produced significantly higher amounts of TGF-β1 at 6 and 24 h (Fig. 8). Compared to vehicle (medium), co-treatment with EP significantly increased the TGF-β1 (24 and 48 h, Fig. 8a) and IL-10 (6 and 48 h, Fig. 8b) production.

### ODE exposure and NF-κB p65 levels

**Fig. 9:**
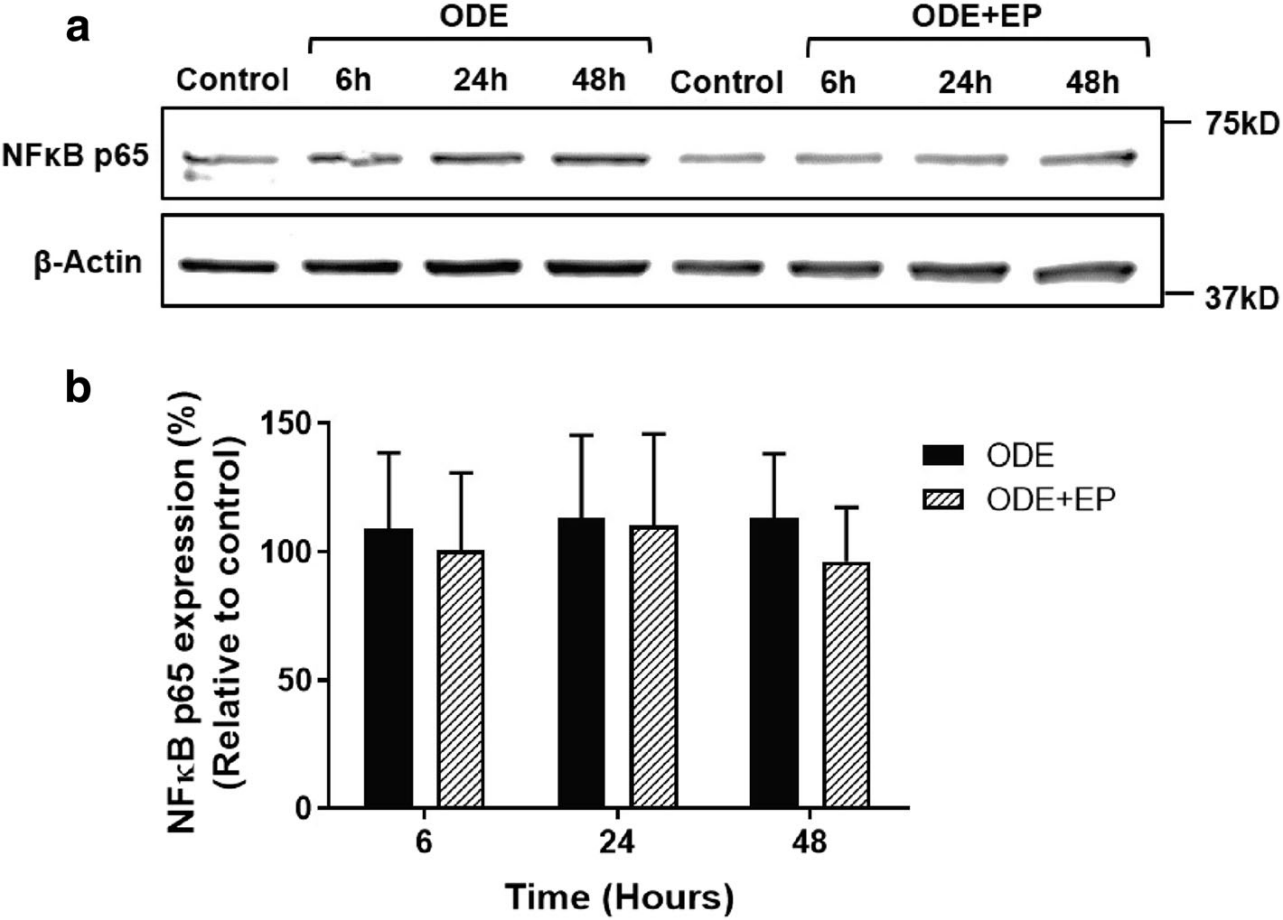
ODE exposure with or without EP-treatment does not alter - NF-κB p65 levels. Medium or ODE treated (with or without co-treatment with EP) whole cell fractions were processed for western blot analysis of NF-κB p65 and β-actin proteins (**a**). Normalized intensity values (as percentage relative to controls) were compared (**b**). There was no difference between any of the treatment groups. Data (*n* = 5) analyzed with one-way ANOVA is represented

We measured the expression of NF-κB p65 levels in the whole cell lysates as a read-out of NF-κB activation upon ODE-exposure (both with and without EP treatment). Normalized densitometry values revealed no difference between control and ODE exposed cells with and without EP treatment at 6, 24 and 48 h (Fig. 9).

### Treatment with EP or neutralizing anti-HMGB1 antibody reduces NF-kB p65 nuclear translocation at earlier time points

**Fig. 10:**
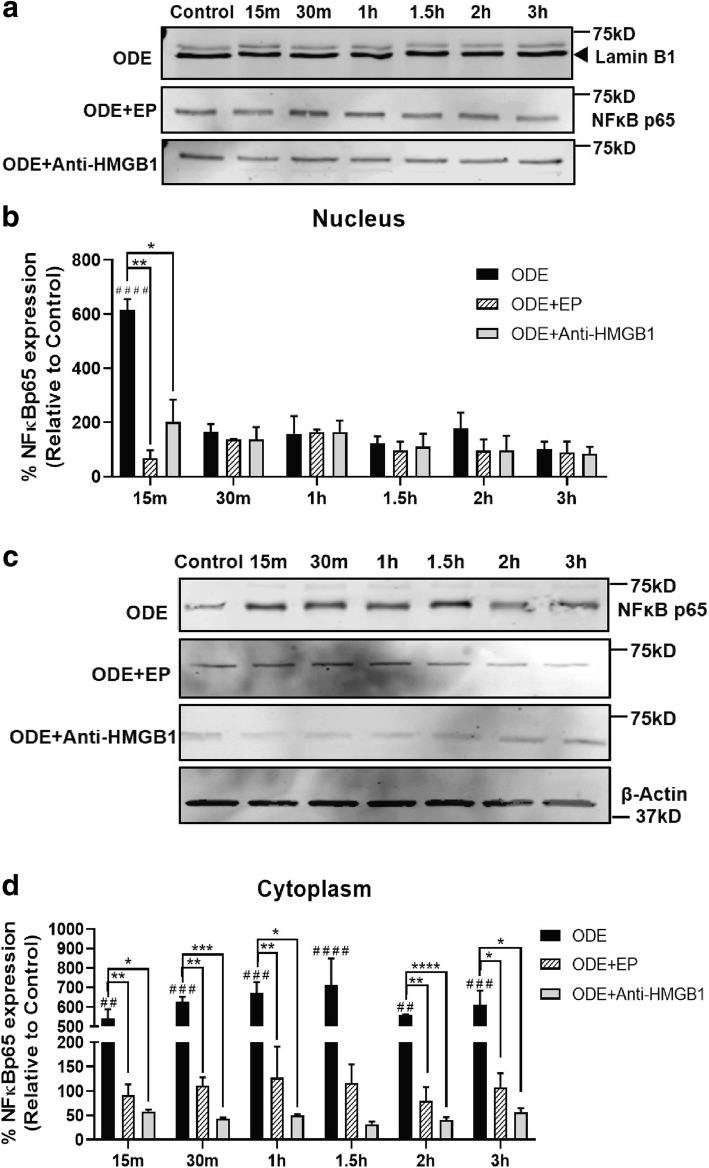
Treatment with EP or anti-HMGB1 neutralizing antibody decreases NF-kB p65 nuclear translocation Cells were processed for separation of nuclear and cytoplasmic fractions and western blotting to detect NF-κBp65 protein. Compared to controls, ODE treated cells showed a temporal increase in NF-κB p65 expression (68 kDa) in the (**a**) nuclear fraction at 15 min. Compared to vehicle (medium), co-treatment with EP and anti-HMGB1 antibody (10 μM) resulted in significant decrease in the levels of NF-κB p65 in the cytoplasm at 15 min post-treatment indicating reduction in ODE-induced NFκB p65 activation (**b** and **d**). NF-κB p65 (68 kD) bands were normalized over either Lamin B1 (50 kD, nuclear fraction, a) or β-actin (37 kD, cytoplasmic fraction, **b**) and percentage intensity (n = 5/group) values of treatment groups relative to control were analyzed using two-way ANOVA (**c** and **d**). * or # *p* < 0.05, ** or ## *p* < 0.01, *** or ### *p* < 0.001, **** or #### *p* < 0.0001. # indicates different from control whereas * indicates difference within the OD/barn exposure groups

We quantified the expression of NF-κB p65 levels in both nuclear and cytoplasmic fractions to delineate the NF-κB activation upon ODE-exposure (both with and without EP or anti-HMGB1 neutralizing antibody treatment). Normalized densitometry values revealed that, compared to controls, ODE exposure increased NF-κB p65 levels in nuclear fractions at 15 and 30 min as well as 1, 1.5, 2 and 3 h. Both EP and anti-HMGB1 antibody treatments significantly decreased the ODE-induced increases in NF-κB p65 nuclear levels at 15 min (Fig. 10, a). Compared to controls, ODE-exposed cells showed increased levels of NF-κB p65 in the cytoplasm at all the time points. Both EP and anti-HMGB1 antibody treatment significantly decreased the levels of NF-κB p65 in the cytoplasmic fractions at 15 and 30 min as well as 1, 2 and 3 h but not at 1.5 h (Fig. 10, b). EP treated cells at 1 and 1.5 h still contained higher amounts of cytoplasmic NF-κB p65 than controls.

### Neutralizing anti-HMGB1 antibody treatment reduces ODE exposure induced secretion of IL-6 but not IL-8 levels

**Fig. 11:**
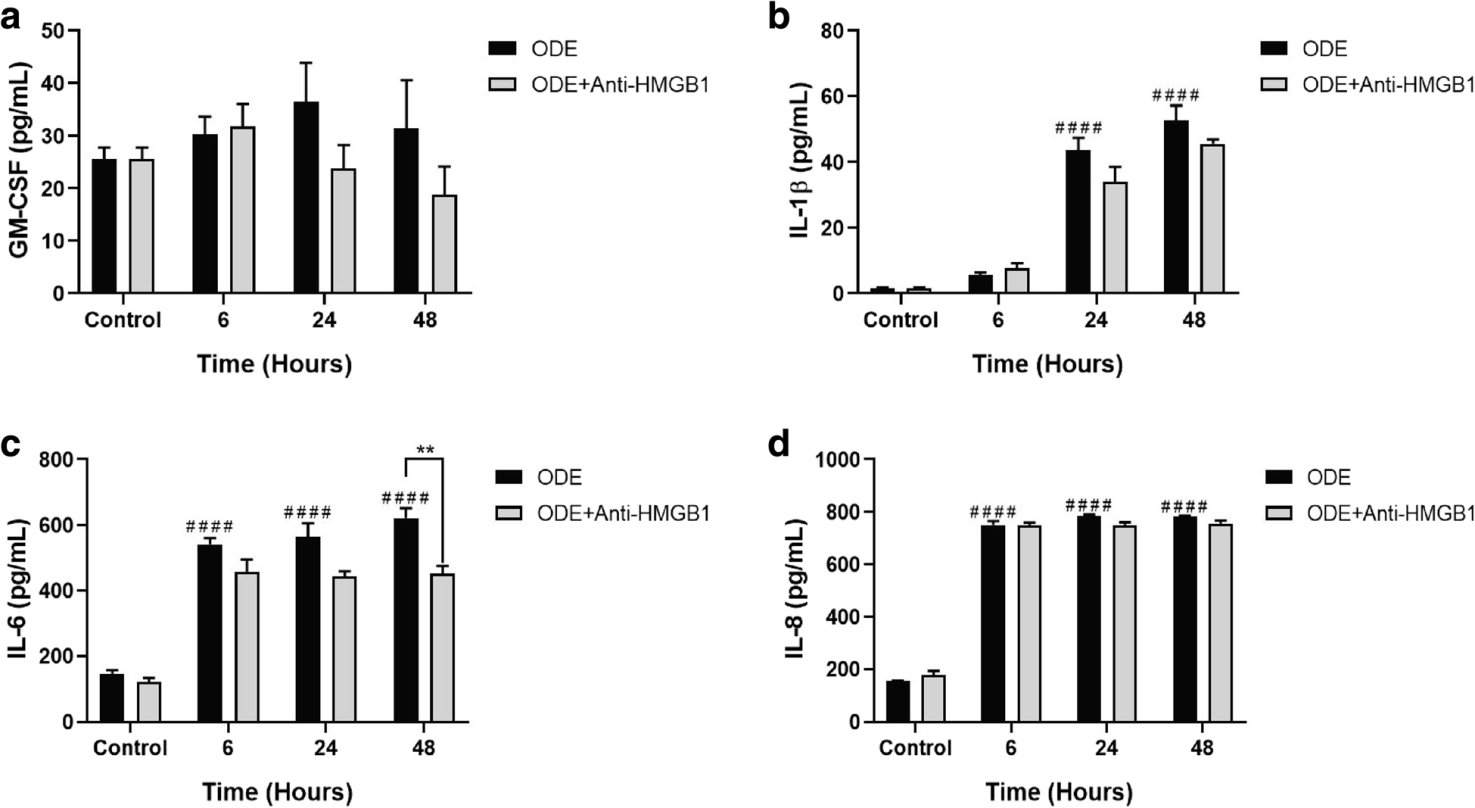
Neutralizing anti-HMGB1 antibody treatment reduces ODE exposure induced secretion of IL-6 but not IL-8 levels. Compared to controls, ODE treatment increased the production of GM-CSF (**a**, 24 and 48 h), IL-1β, IL-6 and IL-8. When ODE exposed cells were treated with anti-HMGB1 antibody (10 μM), significantly reduced ODE-induced increase in IL-6 levels only (**c**). and did not change GM-CSF (**a**), IL-1β (**b**) and IL-8 (**d**) levels. Data (*n* = 6) analyzed using two-way ANOVA is represented. ** or ## *p* < 0.01 and **** or #### *p* < 0.0001. # indicates different from control whereas * indicates difference within the OD/barn exposure groups

Compared to controls, ODE exposure induced an increase in GM-CSF, IL-1β, IL-6 and IL-8 levels. Treatment with neutralizing anti-HMGB1 antibody significantly decreased IL-6 but not IL-1β or IL-8 levels (Fig. 11, a-d).

### Neutralizing ant-HMGB1 antibody treatment augments ODE-induced production of TGF-β1 and IL-10 levels in BEAS-2B cells

**Fig. 12:**
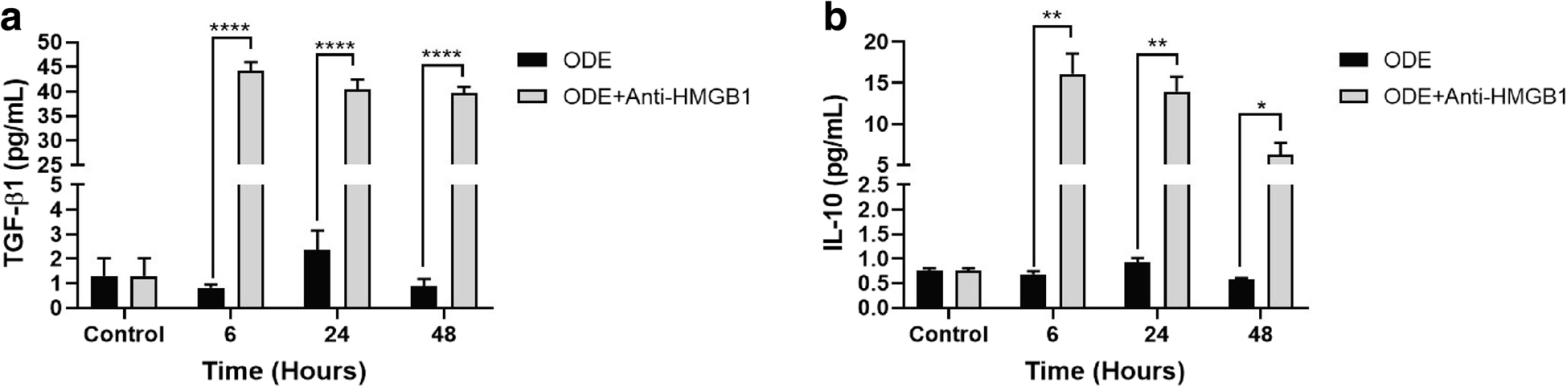
Neutralizing antibody treatment augments ODE-induced production of TGF-β1 and IL-10 levels in BEAS-2B cells. Compared to medium (control, 0 h), co-treatment of ODE exposed BEAS-2B cells with Anti-HMGB1 antibody (10 μM) significantly increased the production of TGF-β1 (**a**, 6, 24 and 48 h) and IL-10 (**b**, 6, 24 and 48 h). Data (*n* = 6) analyzed with two-way ANOVA is represented. * or # *p* < 0.05, ** or ## *p* < 0.01, *** or ### *p* < 0.001, **** or #### *p* < 0.0001. # indicates different from control whereas * indicates difference within the OD/barn exposure groups

Compared to controls, treatment of ODE-exposed cells with anti-HMGB1 neutralizing antibody augmented the production of TGF-β1 and IL-10 at 6, 24 and 48 h (Fig. 12, a and b).

### Neutralizing anti-HMGB1 antibody treatment reduces ODE-induced ROS and nitrite production

**Fig. 13:**
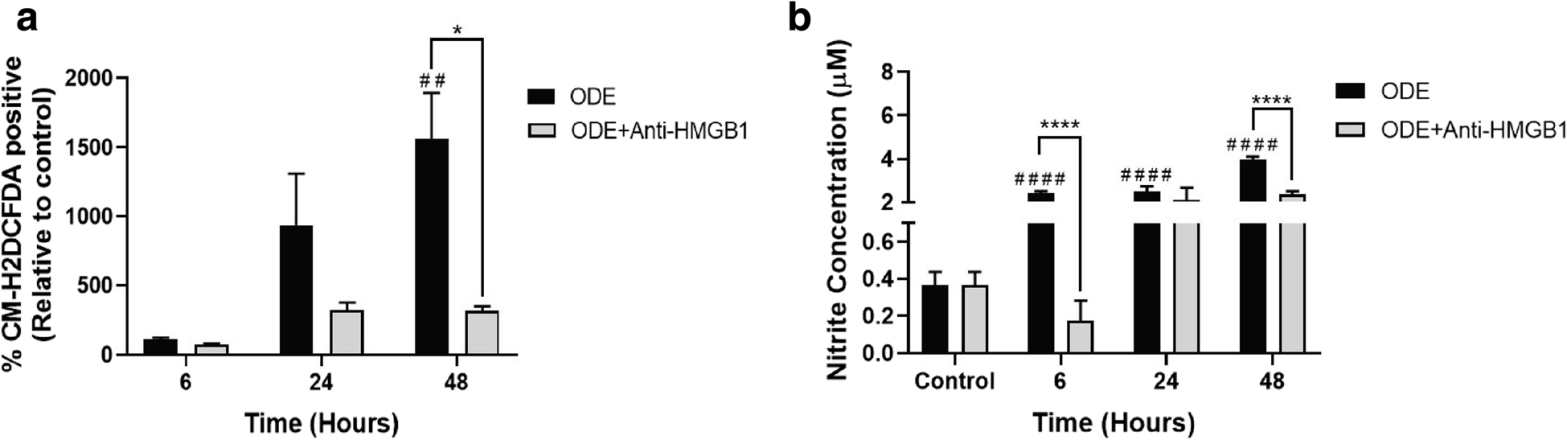
Antibody neutralization treatment reduces ODE-induced ROS and nitrite production. Cells or supernatants were subjected to CM-H2DCFDA and Griess’ assay to quantify intracellular ROS production (**a**) and secreted nitrite concentration (**b**) respectively (*n* = 6). Compared to ODE exposure alone, ODE exposed cells co-treated with Anti-HMGB1 antibody (10 μM) showed a significant reduction in ODE-induced ROS production (48 h) and secreted nitrite (6 and 48 h). Data analyzed with two-way ANOVA is represented (a and b). * or # *p* < 0.05, ** or ## *p* < 0.01, *** or ### *p* < 0.001, **** or #### *p* < 0.0001. # indicates different from control whereas * indicates difference within the OD/barn exposure groups

Compared to controls, ODE exposure induced significant production of intracellular ROS and secreted nitrite (representing RNS). Treatment with neutralizing anti-HMGB1 antibody significantly decreased the intracellular ROS (48 h) and secreted nitrite levels (6 and 48 h, Fig. 13, a and b respectively).

### ODE exposure modulates NF-κB subunit gene expression with time

**Fig. 14:**
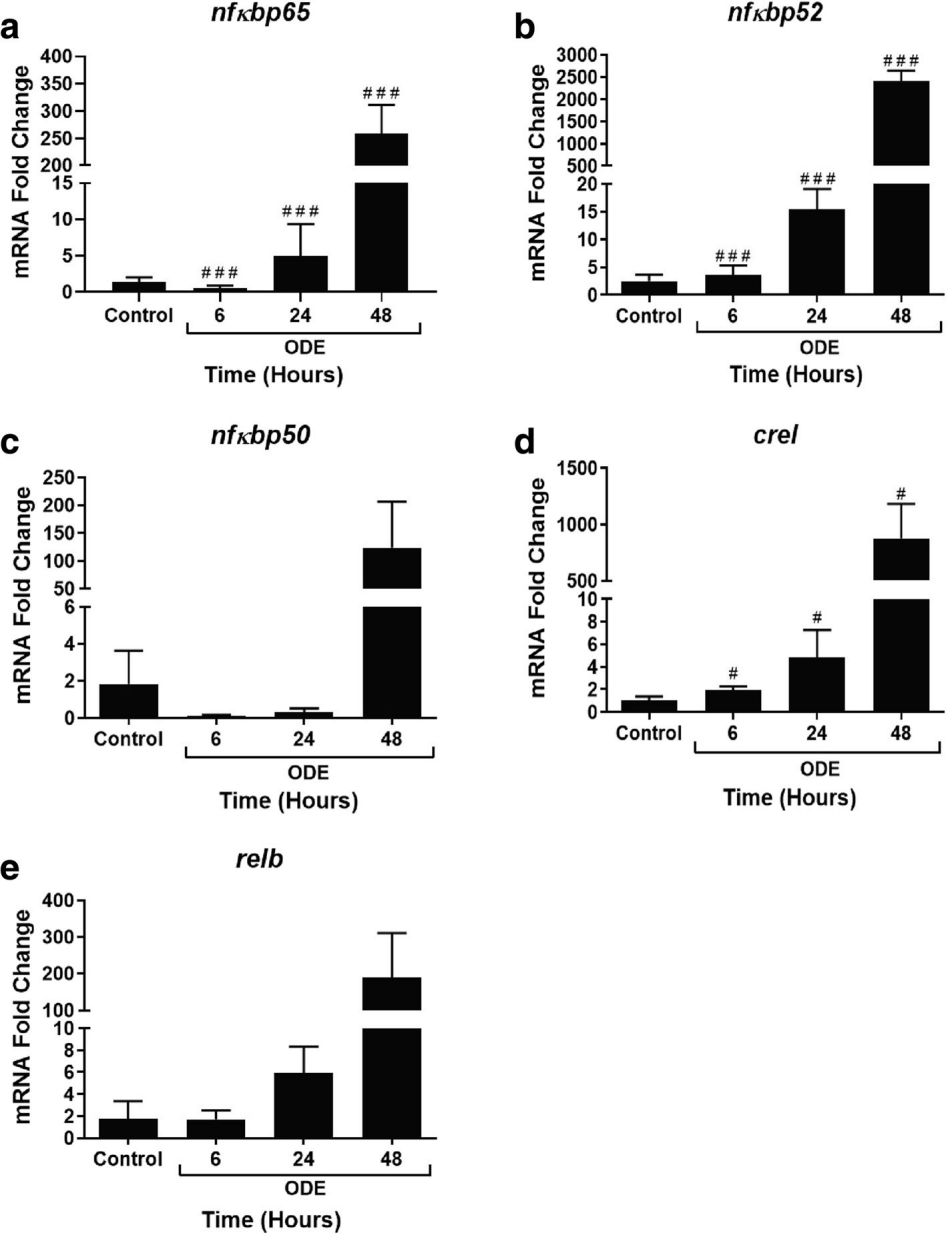
ODE exposure modulates NF-κB subunit gene expression with time. qRT-PCR analysis on NF-κB sub unit genes was performed on control and ODE exposed cells at 6, 24 and 48 h (a-3). Compared controls, ODE-exposure induced a significant increase in *nfkbp65* (**a**), *nfkbp52* (**b**) and *crel* (**d**) at 6, 24 and 48 h (#, *p* < 0.05 and ###, *p* < 0.001 with respect to controls) and did not change *nfkbp50* (**c**) and *relb* (**e**). Data analyzed with one-way ANOVA is represented as fold change of mRNA expression shown relative to untreated control cells

Compared to controls, ODE-exposed cells showed in a significant increase in the transcripts of NF-κB sub units namely, *nf-κbp65, nf-κbp52 and crel* at 6, 24 and 48 h but not *nf-κbp50* and *relb* (Fig. 14, a-e respectively).

### ODE exposure increases tlr2 and tlr4 expression

**Fig. 15:**
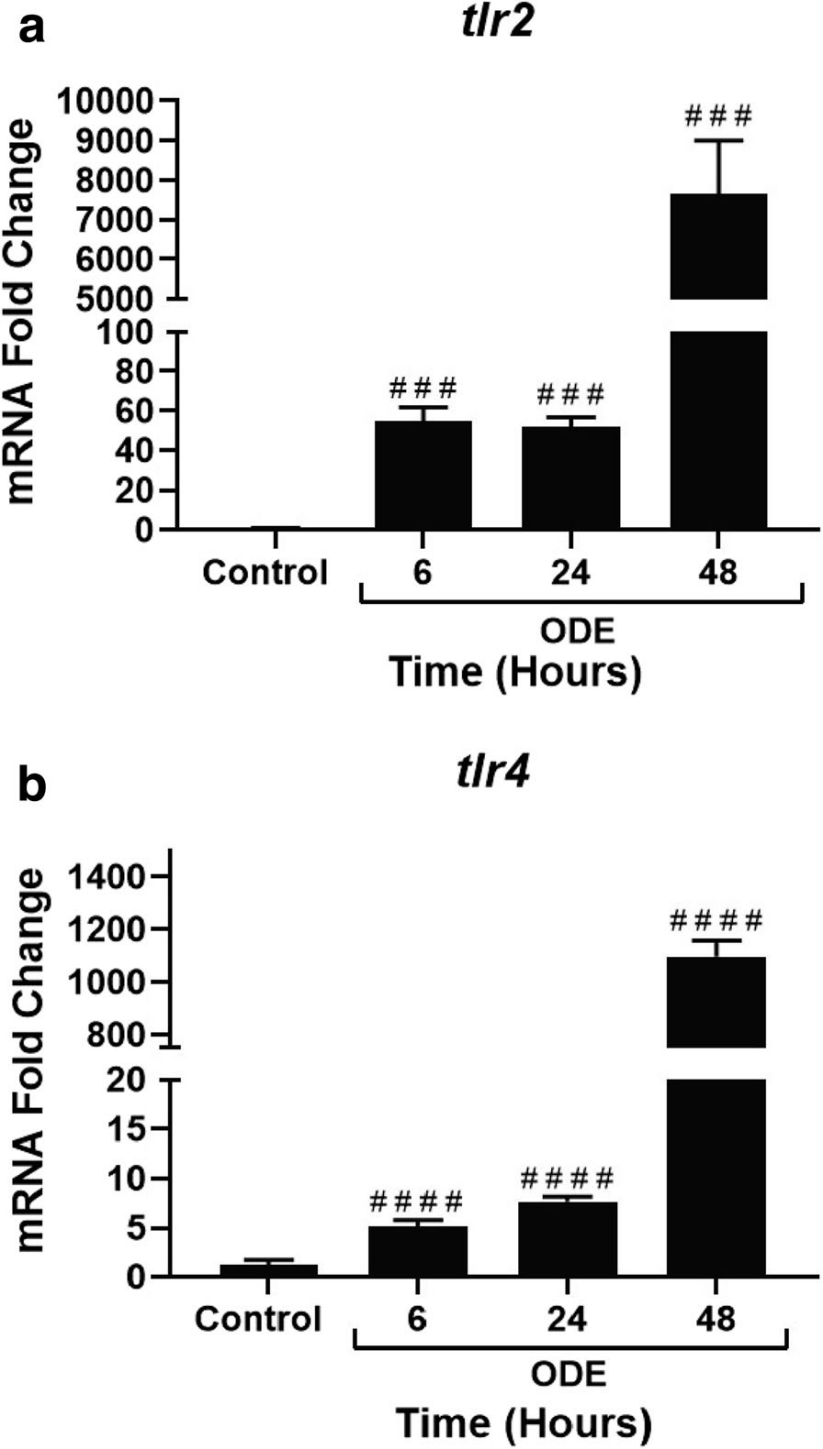
ODE exposure increases *tlr2* and *tlr4* gene expression with time. qRT-PCR analysis on *tlr2* (**a**) and *tlr4* (**b**) genes was performed on control and ODE exposed cells at 6, 24 and 48 h. Compared to controls, ODE exposure resulted in a significant increase in fold in the expression of both *tlr2* and *tlr4* (###, *p* < 0.001 and ####, *p* < 0.0001, **a** and **b** respectively)*.* Data analyzed with one-way ANOVA is represented as fold change of mRNA expression shown relative to untreated control cells

Compared to controls, ODE exposed cells showed a significant increase in the transcripts of *tlr2* and *tlr4* at 6, 24 and 48 h (Fig. 15, a and b).

## Discussion

Persistent exposure to OD is the hallmark of occupational respiratory diseases of agriculture production workers as well as in other industries. Early acute symptoms of airway inflammation dampen over a period of continued exposure, but long-term lung remodeling features and loss of lung function is evident [2, 9]. Unraveling the cell and molecular basis of how low-grade inflammation drives the chronic exposure induced changes may result in better therapies. In this study, we show that OD-exposure of human airway epithelial cells induces nucleocytoplasmic translocation of HMGB1. EP or anti-HMGB1 neutralizing antibody treatment reduces OD-induced inflammation via targeting HMGB1-RAGE pathway.

First, using our well characterized rat model of OD (swine barn) exposure [15], we demonstrated that barn exposure increases expression of HMGB1 in the lung tissue compartments. This indicates that HMGB1 has a possible role in OD-induced lung inflammation and airway reactivity. Other researchers have shown that increased expression of HMGB1 in the airway epithelium leads to pulmonary fibrosis [44] as well as epithelial mesenchymal transition via TGF-β (reviewed in [44, 45]). Next, increased expression of HMGB1 in the lung ASM observed in our study assumes importance since it is known to contribute to ASM dysfunction in a human asthma model [27]. Increased expression of HMGB1 in the blood vessels of the lung is known to cause pulmonary artery hypertension [46] and has a role in ischemia induced blood-brain barrier disruption [47] . Increased expression of HMGB1 in BALT is interesting since HMGB1 is considered to be a central cytokine for all lymphoid cells [48]. Increased expression of HMGB1 in the BALT partly explains our previous observation in the same rat model that a 20-day barn exposure induces activation of BALT [15]. Taken together, increased expression of HMGB1 in the lung following 1, 5, and 20-day barn exposure indicates the potential pathological role of this secreted DAMP molecule.

Using in vitro model of human airway exposure to OD, we now demonstrate nucleocytoplasmic translocation of HMGB1, increased expression as well as cytoplasmic co-localization of HMGB1 with RAGE in the cytoplasm. Instead of normal nuclear location, accumulation of HMGB1 in the cytoplasm indicates cellular stress and cytoplasmic HMGB1 is a chief regulator of autophagy [49]. Secreted HMGB1 is known to potentiate inflammation in the presence of PAMPs such as LPS [50]. Our results indicate that ODE-exposure induced HMGB1 accumulation in the cytoplasm and secretion into extracellular compartment particularly in chronic exposure to OD may drive aberrant airway inflammation and lead to lung remodeling features [50]. Although, our efforts to quantify secreted HMGB1 were not fully successful, we did observe higher molecular weight bands for secreted HMGB1 in the cell culture supernatant (data not shown). Posttranslational modifications such as acetylation and oxidation of HMGB1 may have contributed to this higher molecular weight HMGB1. Currently, few laboratories have expertise and established methods to measure secreted HMGB1 with post-translational modification/s.

Immunocytochemistry showed co-localization of HMGB1 and RAGE in the cytoplasm indicated possible physical interaction of HMGB1 with its main receptor RAGE. To our knowledge, this is possibly the first report of secretion of DAMP in an in vitro model of human airway epithelial cell exposure to OD and co-localization of HMGB1 with RAGE. It is likely that increased expression and secretion of HMGB1 (cytoplasmic and extracellular) may be driving a sustained inflammation in long-term OD-exposed individuals via HMGB1-RAGE signaling.

Next, using EP treatment we were able to abrogate ODE-induced nucleocytoplasmic translocation of HMGB1. EP treatment also decreased the expression and co-localization of HMGB1 and RAGE in the cytoplasm. EP is known to prevent phosphorylation of HMGB1 by chelating calcium and thereby prevents nucleocytoplasmic translocation of HMGB1 [36]. Other post-translational modifications such as acetylation have been shown to be important for nucleocytoplasmic translocation of HMGB1 [51]. Our study did not examine if any of the posttranslational modification were involved in OD-induced translocation of HMGB1. Our central focus was to examine if nucleocytoplasmic translocation and secretion of HMGB1 would be influencing OD-induced airway inflammation.

We have demonstrated that ODE exposure of airway epithelium results in ROS and RNS production. When we arrested nucleocytoplasmic translocation of HMGB1 using EP treatment, we observed significantly decreased levels of ROS but not RNS. It is interesting to note that, ODE exposure increased the ROS levels at 24 and 48 h whereas RNS production was significantly higher starting from 6 h onwards. Based on our results, it is possible that ODE-induced translocation of HMGB1 has a role in potentiating ROS but not RNS levels. This is again supported by the fact that EP mediated arrest of HMGB1 translocation did not affect ODE-induced RNS levels at all the time points. Though we do not provide direct mechanistic evidences, these results pave way for further investigation.

To examine if HMGB1 translocation had any effect on inflammatory mediators, we measured the levels of GM-CSF, IL-1β and IL-6 (pro-inflammatory), TGF-β1 (pleiotropic cytokine) and IL-10 (anti-inflammatory) and IL-8 (neutrophil chemokine). ODE-exposure increased the levels of GM-CSF, IL-1β, IL-6 and IL-8. Secretion of GM-CSF by airway epithelial cells is important since it is known to sensitize the airway epithelium to allergic insults such as house dust mite or cockroach allergen [52]. Further, GM-CSF in an inflammatory tissue milieu which acts as a link between recruited lymphocytes and monocytes and could be targeted to reduce chronic inflammation [53]. Next, our observation of increase in IL-1β production is consistent with published work on BEAS-2B cells exposed to poultry barn dust extract [54]. IL-1β has a variety of inflammatory effects in the lung including induction of airway hyper responsiveness [55, 56]. ODE-induced IL-6 production assumes significance since the recent work has identified IL-6 as an important link in lung-bone inflammatory axis in a mouse model of intra-nasal ODE exposure [21]. Next, IL-8 secretion observed in our study is in line with other published evidences [57, 58]. IL-8 is known to attract neutrophils to the site of inflammation and neutrophilic oxidative stress upon exposure to OD is known to drive pulmonary inflammation and airway reactivity [59]. Using a specific antagonist against IL-8 was beneficial in an OD-induced lung inflammation model [60].

Lastly, we demonstrated that EP-treatment augments IL-10 and TGF-β production in ODE-exposed airway epithelial cells. Production of IL-10 generally results in anti-inflammatory effects (reviewed in [61]). Airway epithelial cell derived TGF-β is important in driving type 2 innate lymphoid cell responses to allergens and TGF-β is known to exacerbate house dust mite-induced pathology and increased expression of TGF-β is seen with viral and allergen challenge (reviewed in [62]). Taken together, upon ODE exposure, human airway epithelial cells secrete a variety of inflammatory mediators.

Our unique observation that EP treatment had no effect on the ODE-induced IL-8 and IL-6 indicates several possibilities. First, HMGB1 mediated signaling pathway as well as ODE-induced IL-8/IL-6 secretion may be independent of each other. Second, IL-8 and IL-6 production peaks from 6 h after ODE-exposure and it is likely that secreted HMGB1 acts at a later time point to potentiate the inflammation. Although we do not provide any direct evidence, there is a link between decrease in cytoplasmic/secreted HMGB1 levels following EP treatment and decrease in ODE-induced GM-CSF and IL-1β levels. This again highlights the fact that HMGB1 is a late-player in many inflammatory events (reviewed in [63]).

In order to examine mechanistically, if arrest of nucleocytoplasmic translocation of HMGB1 reduces inflammatory mediators through NF-κB, immunoblots for NF-κB p65 in control (0 h) and ODE exposure (6, 24 and 48 h) with media or EP treatments revealed no significant difference between any of the groups (all time points). However, when we probed the NF-κB p65 levels in the nuclear and cytoplasmic fractions at early time points, we found that ODE exposure increases both nuclear and cytoplasmic levels of NF-κB p65 as early as 15 min. Both EP and anti-HMGB1 antibody treatments significantly reduced NF-κB p65 levels in both nucleus and cytoplasm indicating that their anti-inflammatory action is via a reduction in the translocation of NF-κB p65.

We and others have previously shown the roles of TLR2 [18] and TLR4 [16] in OD exposure induced lung inflammation. We now show significant increase in the expression of *tlr2* and *tlr4* genes following ODE exposure. We observed translocation of NF-κB p65 into the nucleus as early as 15 min indicating that ODE-induced innate signaling via TLR2 and 4 results in NF-κB activation leading to cytokine secretion. However, both EP and anti-HMGB1 antibody treatments inhibited both nuclear translocation of NF-κB p65 and pro-inflammatory cytokine production to highlight the importance of targeting HMGB1. Since pharmacological tool blocking HMGB1 translocation and RAGE expression (EP) or genetic tool suppressing the expression of HMGB1 is able to reduce ODE-induced inflammation, HMGB1 signaling is a major driver of ODE-induced airway inflammation. Though our study has not investigated the specific downstream events in this pathway, EP or anti-HMGB1 antibody mediated targeting of HMGB1-RAGE pathway is promising.

## Conclusions

OD exposure of human airway epithelial cell line results in the nucleocytoplasmic translocation of HMGB1, cytoplasmic co-localization of HMGB1 with RAGE as well as production of ROS, RNS and inflammatory cytokines. EP or anti-HMGB1 antibody treatment abrogates OD exposure induced translocation of HMGB1 and many inflammatory end-points. Therefore, HMGB1-RAGE signaling is an attractive target to treat OD-induced occupational lung diseases.

## Abbreviations

ASM: Airway smooth muscle
BALT: Bronchus associated lymphoid tissue
CAFOs: Concentrated animal feeding operations
DAMPs: Damage associated molecular patterns
EP: Ethyl pyruvate
HMGB1: High mobility group box-1
LPS: Lipopolysaccharide
NLS: Nuclear localization sites
OD: Organic dust
ODE: Organic dust extract
PAMPs: Pathogen associated molecular patterns
PGN: Peptidoglycan
RAGE: Receptor for advanced glycation end products
